# The role of serum albumin and albumin-related nutritional indices in predicting post-stroke cognitive impairment: a systematic review and meta-analysis

**DOI:** 10.3389/fneur.2025.1641711

**Published:** 2025-08-13

**Authors:** Yan-qiu Wang, Xia He, Xia-lian Huang, Fu-li Qin, Feng-le Mao, Yue-ming Cheng, Xiao-xue Zeng, Ying-ying Yang, Ming-xi Xu

**Affiliations:** ^1^School of Health Preservation and Rehabilitation, Chengdu University of Traditional Chinese Medicine, Chengdu, China; ^2^Department of Neurology, Affiliated Sichuan Provincial Rehabilitation Hospital of Chengdu University of Traditional Chinese Medicine, Chengdu, China

**Keywords:** stroke, cognitive impairment, serum albumin, nutritional index, predictive biomarkers

## Abstract

**Background:**

The role of serum albumin levels and albumin-related nutritional indices in the prediction of cognitive impairment after stroke has not reached a uniform conclusion.

**Methods:**

This study was prospectively registered in PROSPERO (CRD420251012150) and followed the PRISMA guidelines. We systematically searched six databases with a time frame from the date of database establishment to March 29, 2025. Literature selection and data collection were conducted by two researchers. Assessment of literature quality was performed according to the Newcastle-Ottawa Scale (NOS). Weighted mean difference (WMD) with 95% confidence intervals (CIs) was used to express pooled effect sizes. The chi-square (χ2) test (Cochran’s Q) and index of inconsistency (*I*^2^) were used to detect heterogeneity.

**Results:**

A total of 9 studies involving 2,332 stroke patients were included in this meta-analysis. The results of this study showed that serum albumin levels (WMD: −3.85; 95% CI: −5.61, −2.09; *p* < 0.0001), Geriatric nutritional risk index (GNRI) (WMD: −2.68; 95% CI: −4.97, −0.39; *p* = 0.02), and HALP (hemoglobin, albumin, lymphocyte, and platelet) scores (WMD: −10.74; 95% CI: −19.98, −1.50; *p* = 0.02)were significantly lower in the post-stroke cognitive impairment (PSCI) compared to the post-stroke non-cognitive impairment (PSNCI).

**Conclusion:**

Decreased serum albumin levels and albumin-related nutritional indices (GNRI and HALP scores) have a strong correlation with PSCI, which may become important indicators for early prediction of the development of PSCI.

**Systematic review registration:**

https://www.crd.york.ac.uk/prospero/#recordDetails, identifier, CRD420251012150.

## 1 Introduction

Stroke, the most common cause of mortality worldwide, has imposed a huge economic burden on society due to its increasing incidence, high mortality, and disability rates (1). Post-stroke cognitive impairment (PSCI) is a complication of stroke characterized by memory loss, visuospatial deficits, and decreased executive functioning (2, 3). It will lead to irreversible cognitive impairment if not intervened on promptly, which can severely impact a patient’s quality of life and longevity (4). The study suggests that the period between stroke occurrence and the development of PSCI is a critical treatment window for the prevention of cognitive impairment (5). Accordingly, it is essential to identify predictive indicators for effective screening of people at risk for PSCI at early stages so as to improve the quality of patients.

The rates of malnutrition after stroke can be as high as 62% (6). Research has shown a strong association between nutritional condition and cognitive performance, and a favorable state of nutrition is crucial for the maintenance and improvement of cognitive performance (7, 8). Serum albumin is one of the major proteins in the blood with functions in maintaining nutrition and osmolality in the body, and is often used as an important indicator of a patient’s nutritional status (9, 10). Studies indicated that serum albumin can improve cerebral circulation and has a protective effect on both neurons and glial cells (11). Decreased serum albumin levels also indicate poorer functional prognosis after stroke (12, 13). In recent years, albumin-related nutritional indices, such as the Geriatric Nutritional Risk Index (GNRI) and the HALP (hemoglobin, albumin, lymphocyte, and platelet) scores, have been increasingly used in cancer prognosis (14, 15). However, it is unclear whether these indices are relevant to PSCI. Consequently, the purpose of this research was to evaluate the value of serum albumin levels and albumin-related nutritional indices in the prediction of PSCI by providing evidence for their application in the prevention and intervention of cognitive impairment after stroke.

## 2 Methods

This meta-analysis identified the predictive value of albumin-related nutritional indicators for PSCI. The study protocol was registered in PROSPERO (CRD420251012150) and conducted according to the PRISMA guidelines. The PRISMA 2020 checklist can be found in Supplementary Table S1.

### 2.1 Search strategy

We searched PubMed, Embase, Cochrane Library, Web of Science, China National Knowledge Infrastructure (CNKI), and Wanfang databases systematically, with a search timeframe from database creation to March 29, 2025. In addition, we manually searched other literature that might meet the inclusion criteria. The search terms are shown in Supplementary Table S2.

### 2.2 Criterion of inclusion and exclusion

#### 2.2.1 Inclusion criteria

(1) The participants in the study were patients who suffered or did not suffer from any cognitive impairment after stroke; (2) Target predictors included serum albumin levels and albumin-derived composite indices (specifically GNRI and HALP); (3) observational studies.

#### 2.2.2 Exclusion criteria

(1) Reviews, meta-analysis, conference, case report, duplicate literature, dissertations, animal experiments; (2) full text or complete data unavailable; (3) studies not in English or Chinese.

### 2.3 Literature selection and data extraction

Researchers (YQW, FLQ) independently completed the literature screening and data extraction process. When disagreements occurred, they were discussed with the researcher (XH) until a consensus was reached. When the continuous variable in the study was reported as median, we calculated means±standard deviations (SD) by using methods that have been validated (16, 17). If the data was incomplete, we tried to contact the authors by email to acquire the complete data.

### 2.4 Quality assessment of literature

Two researchers (YQW, XLH) conducted the quality assessment of the included literature by using the Newcastle-Ottawa Scale (NOS). This scale is scored out of nine points and consists of three dimensions: selection, comparability, and exposure/outcome. Six or more points indicate high-quality research.

### 2.5 Statistical analysis

We used Review Manager 5.4 for statistical analysis. Continuous variables were analyzed using weighted mean difference (WMD) with 95% confidence intervals (CI) as the statistic for effect analysis. The chi-square (χ^2^) test (Cochran’s Q) and the inconsistency index (*I* ^2^) were used to evaluate the degree of heterogeneity among the studies. Fixed-effects model (*p* > 0.05 or *I* ^2^ ≤ 50%) or random-effects model (*p* ≤ 0.05 or *I* ^2^ > 50%) were used to conduct a meta-analysis. The pooled estimates were displayed using the forest plot.

### 2.6 Subgroup analysis

Subgroup analysis was conducted based on the type of study design, cognitive assessment scale, time of blood tests, and the time experienced after stroke onset.

### 2.7 Sensitivity analysis

We evaluated the impact of each research on the pooled effect under conditions of significant heterogeneity by the leave-one-out method.

## 3 Results

### 3.1 Literature search and study characteristics

**Figure 1:**
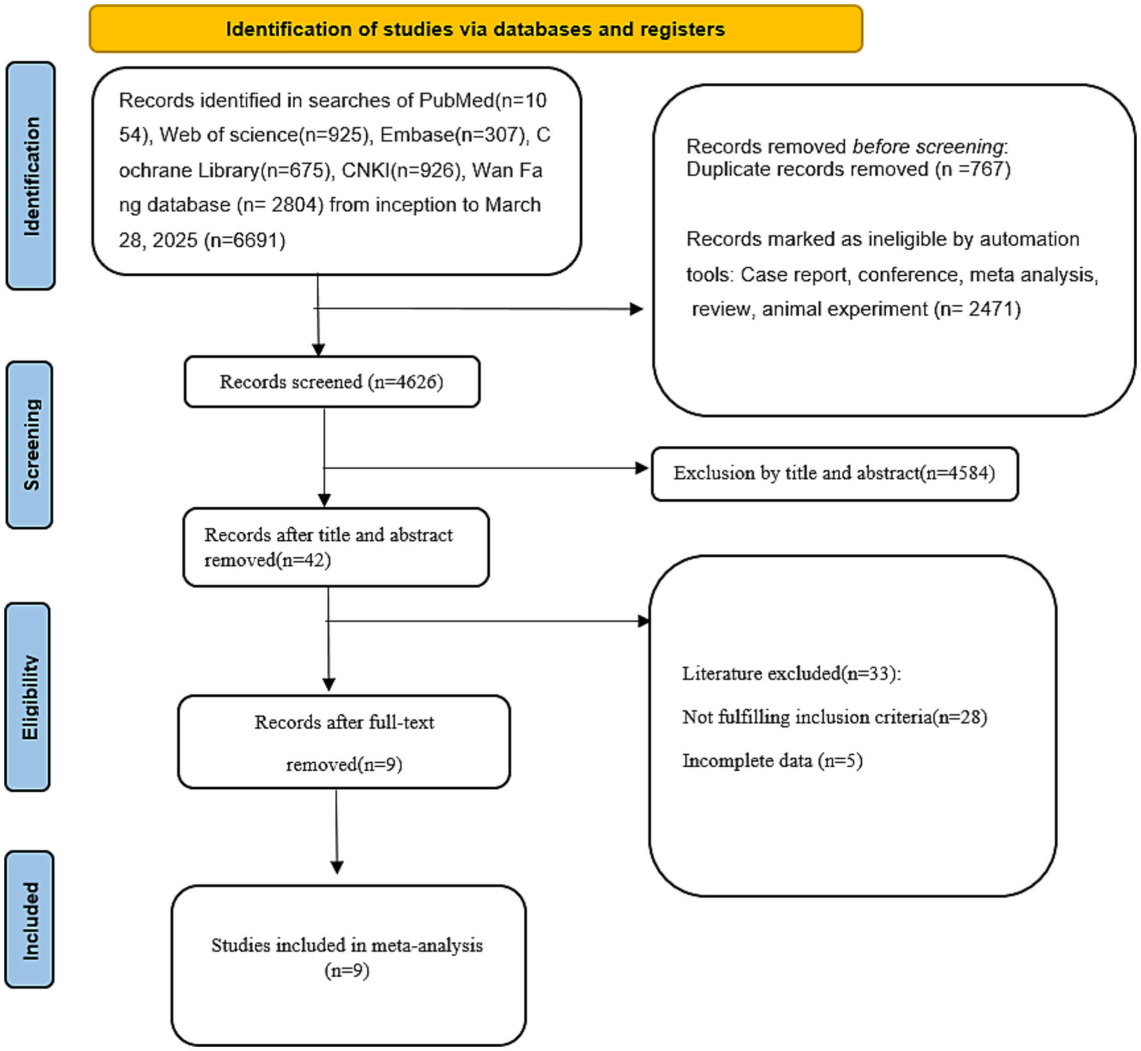
PRISMA flow chart of literature searching and screening.

There were 6,691 articles that we retrieved. By using the automatic screening tool of EndNote (version 2020) software, 3,238 studies were excluded. We excluded 4,584 studies by reading the titles and abstracts, leaving 42 relevant studies for full-text reading. Ultimately, a total of 9 studies (8, 10, 18–24) involving 2,332 patients were included through rigorous screening. The process of literature selection is shown in Figure 1. Among all the included studies, only one (20) was from Korea, and the other eight (8, 10, 18, 19, 21–24) were from China. In terms of type of study design, there were three retrospective cohort studies (8, 10, 18), two case–control studies (19, 23), and four prospective cohort studies (20–22, 24). The basic information of the included studies is presented in Table 1. All included studies were assessed as low risk of bias according to the Newcastle-Ottawa Scale (NOS), with scores ≥ 6. Specifically, cohort studies (n = 6) scored 6–9 points, and case–control studies (n = 3) scored 7–9 points. No studies were classified as low-quality (NOS < 6). Detailed NOS evaluations are presented in Table 2.

**Table 1:** Characterization of the studies included in the systematic review.

Author/year	Country	Study design	Cognitive assessment	Time of blood test	Time of stroke onset	PSCI group					
						Sample	Age	Male	ALB	GNRI	HALP
Shuen Li 2020 (18)	China	Retrospective cohort	MMSE	Within 18 h after admission	Onset within 72 h	124	67.5 ± 11.3	82	35.3 ± 4.6		
Minwoo Lee 2021 (20)	Korea	Prospective cohort	MMSE	after admission	Onset within 1 week	70	66.9 ± 11.3	37		102.9 ± 9.6	
Hui Men 2021 (19)	China	Case–control	MMSE	The next day after admission	Onset within 2 weeks	48	68.6 ± 11.6	30	39.84 (35.49,42.58)^*^		
Mingming Gao 2022 (21)	China	Prospective cohort	MMSE MoCA	NA	NA	38	64.3 ± 4.2	22	42.3 ± 4.6		
Yanbin Li 2022 (22)	China	Prospective cohort	MMSE	The next day after admission	Onset within 48 h	56	66.6 ± 5.3	33	38.5 ± 3.54		
Minjie Xu 2023 (10)	China	Retrospective cohort	MMSE	Within 24 h after admission	Onset within 1 week	382	68.0 (60.8, 74.0)^*^	229	37.6 (35.5, 39.5)^*^		38.7 (28.6, 52.1)^*^
Zhiqing Cheng 2023 (23)	China	Case–control	MoCA	NA	Onset within 1 week	51	63.47 ± 11.38	29	39.74 ± 4.69		
Tao Zhou 2024 (24)	China	Prospective cohort	MoCA	The next day after admission	Onset within 1 week	106	64.13 ± 12.31	69	38.85 ± 4.29		33.35 (27.30, 49.93)^*^
Yongchun Wang 2024 (8)	China	Retrospective cohort	MMSE	Within 24 h after admission	NA	121	63.0 (58.0, 70.0)^*^	77	37.7 (36.1, 39.4)^*^	102 (97,107)^*^	

Author/year	PSNCI group					
	Sample	Age	Male	ALB	GNRI	HALP
Shuen Li 2020 (18)	284	62.7 ± 12.5	203	38.6 ± 3.5		
Minwoo Lee 2021 (20)	274	62.0 ± 12.0	184		106.9 ± 8.1	
Hui Men 2021 (19)	55	59.4 ± 10.0	41	42.60 (38.85, 44.80)^*^		
Mingming Gao 2022 (21)	62	56.8 ± 6.5	40	53.3 ± 5.2		
Yanbin Li 2022 (22)	89	65.4 ± 6.6	54	44.4 ± 4.54		
Minjie Xu 2023 (10)	210	63.0 (54.0, 69.0)^*^	149	38.4 (36.3, 40.6)^*^		45.4 (33.4, 59.2)^*^
Zhiqing Cheng 2023 (23)	84	58.38 ± 9.37	51	45.48 ± 6.37		
Tao Zhou 2024 (24)	114	57.72 ± 11.09	91	40.64 ± 3.03		52.62 (41.01, 64.35)^*^
Yongchun Wang 2024 (8)	164	61.0 (55.0, 67.0)^*^	124	38.1 (36.3, 40.1)^*^	104 (98,109)^*^	

^*^Median[range]; NA, not applicable; MMSE, Minimum Mental State Examination; MoCA, Montreal Cognitive Assessment; PSCI, Post-stroke cognitive impairment; PNSCI, post-stroke non-cognitive impairment; ALB, albumin; GNRI, Geriatric Nutritional Risk Index; HALP, hemoglobin, albumin, lymphocyte, and platelet.

**Table 2:** Risk of bias assessment according to the Newcastle-Ottawa Scale.

Reference	Study design	Selection	Comparability	Exposure/outcome	Total
Shuen Li 2020 (18)	Retrospective cohort	****	*	***	8
Minwoo Lee 2021 (20)	Retrospective cohort	****	*	***	8
Hui Men 2021 (19)	Case–control	***	*	***	7
Mingming Gao 2022 (21)	Prospective cohort	****	*	**	7
Yanbin Li 2022 (22)	Prospective cohort	***	*	***	7
Minjie Xu 2023 (10)	Retrospective cohort	***	*	**	6
Zhiqing Cheng 2023 (23)	Retrospective cohort	****	**	**	8
Tao Zhou 2024 (24)	Case–control	****	**	***	9
Yongchun Wang 2024 (8)	Prospective cohort	****	*	***	8

^*^1 point, ^**^2 points, ^***^3 points, ^****^4 points.

### 3.2 The results of meta-analysis

#### 3.2.1 Association of serum albumin levels with PSCI

**Figure 2:**
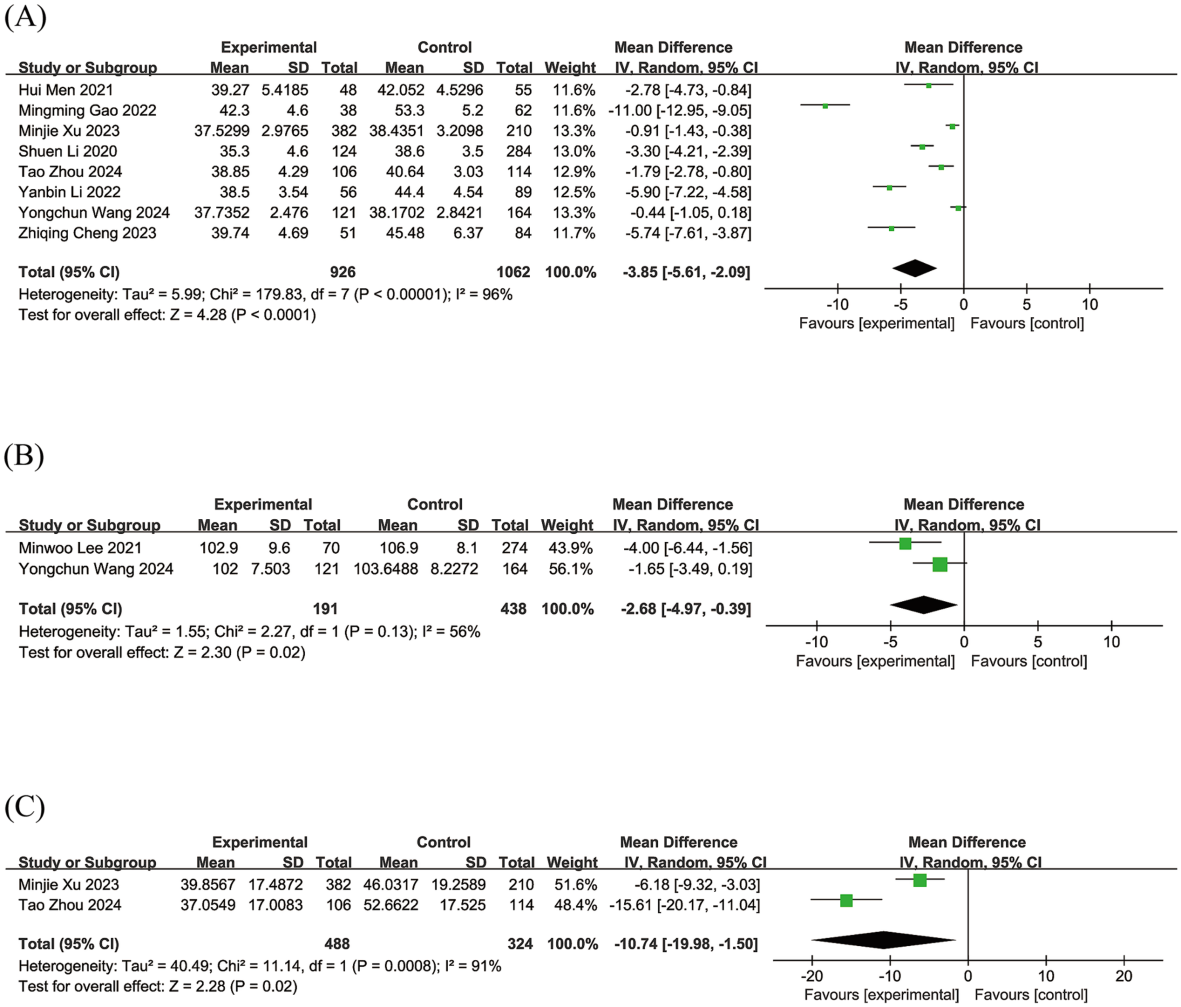
Forest plots of **(A)** serum albumin levels, **(B)** GNRI scores, and **(C)** HALP scores.

A total of eight studies reported the association between serum albumin levels and PSCI. High heterogeneity existed between studies (I^2^ = 96%, *p* < 0.00001), so a random-effects model was applied. Our findings suggested that serum albumin levels were markedly lower in patients with PSCI compared to post-stroke non-cognitive impairment (PSNCI) (WMD = −3.85; 95% CI: −5.61, −2.09, *p* < 0.00001) (Figure 2A).

#### 3.2.2 Association of GNRI scores with PSCI

Two studies compared the GNRI scores between the PSCI and PSNCI groups. Because of the high heterogeneity between studies (*I^2^* = 56%, *p* = 0.13), we used a random-effects model. The results showed that the GNRI scores were notably lower in PSCI patients than PSNCI patients (WMD = −2.68; 95% CI: −4.97, −0.39, *p* = 0.02) (Figure 2B).

#### 3.2.3 Association of HALP scores with PSCI

There were also two studies comparing the PSCI group with the PSNCI group on HALP scores. We used the random effects model to conduct our analysis (*I^2^* = 91%, *p* = 0.0008). Our study showed that patients with PSCI had lower HALP scores compared to PSNCI (WMD: -10.74; 95% CI: −19.98, −1.50; *p* = 0.02) (Figure 2C).

### 3.3 Subgroup analysis

Subgroup analyses specifically focused on studies reporting an association between serum albumin and PSCI. These analyses demonstrated consistent reductions in serum albumin among PSCI patients across study designs, with statistically significant effects observed in prospective cohort studies (WMD = −6.17, 95% CI: −11.01, −1.33; *p* = 0.01), case–control studies (WMD = −4.27, 95% CI: −7.17, −1.37; *p* = 0.004), and retrospective cohort studies (WMD = −1.50, 95% CI: −2.93, −0.08; *p* = 0.04) (Supplementary Figure S1).

When stratified by cognitive assessment tool (Minimum Mental State Examination (MMSE) scale and Montreal Cognitive Assessment (MoCA) scale), the MMSE subgroup showed a significant association between lower serum albumin and PSCI (WMD = −2.59, 95% CI: −4.26, −0.91; *p* = 0.002). In contrast, the MoCA subgroup revealed no statistically significant association (WMD = −3.68, 95% CI: −7.55, 0.19; *p* = 0.06). Although the MMSE+MoCA subgroup (single study) reported a marked albumin reduction (WMD = −11.00, 95% CI: −12.95, −9.05; *p* < 0.00001), this finding requires cautious interpretation due to limited evidence (Supplementary Figure S2).

Analyses based on blood collection timing further indicated significant albumin reductions in PSCI patients when blood was drawn ≤48 h after admission (WMD = −2.44, 95% CI: −3.83, −1.05; *p* = 0.0004). The unlimited timing group also showed a significant reduction (WMD = −8.36, 95% CI: −13.52, −3.21; *p* = 0.001) (Supplementary Figure S3).

Finally, stratification by time of stroke onset revealed significant albumin reductions in acute (≤72 h; WMD = −4.55, 95% CI: −7.10, −2.01; *p* = 0.0005) and early subacute phases (≤1 week; WMD = −2.59, 95% CI: −4.64, −0.55; *p* = 0.01), while the late subacute phase (≤2 weeks; single study) showed significantly lower levels (WMD = −2.78, 95% CI: −4.73, −0.84; *p* = 0.005). No significant association emerged in the unlimited onset time group (WMD = −5.68, 95% CI: −16.03, 4.68; *p* = 0.28) (Supplementary Figure S4). All results of the subgroup analyses are summarized in Table 3.

**Table 3:** Subgroup analysis for association between ALB and PSCI.

Subgroup	ALB			
	*N*	WMD (95%CI)	*p* value	I^2^
Total	8	−3.85 (−5.61, −2.09)	*p* < 0.00001	96%
Study design				
Retrospective cohort study	3	−1.50 (−2.93, −0.08)	*p* = 0.04	93%
Prospective cohort study	3	−6.17 (−11.01, − 1.33)	*p* = 0.01	97%
Case–control study	2	−4.27 (−7.17, −1.37)	*p* = 0.004	78%
Cognitive assessment				
MMSE	5	−2.59 (−4.26, −0.91)	*p* = 0.002	95%
MoCA	2	−3.68 (−7.55, 0.19)	*p* = 0.06	93%
MMSE + MoCA	1	−11.00 (−12.95, −9.05)	*p* < 0.00001	-
Time of blood test				
≤48 h	6	−2.44 (−3.83, −1.05)	*p* = 0.0006	93%
Unlimited	2	−8.36 (−13.52, −3.21)	*p* = 0.001	93%
Time of stroke onset				
≤72 h	2	−4.55 (−7.10, −2.01)	*p* = 0.0005	90%
≤1 week	3	−2.59 (−4.64, −0.55)	*p* = 0.01	92%
≤2 weeks	1	−2.78 (−4.73, −0.84)	*p* = 0.005	-
Unlimited	2	−5.68 (−16.03, 4.68)	*p* = 0.28	99%

*N*, number; ALB, albumin; WMD, weighted mean difference; CI, confidence interval.

### 3.4 Sensitivity analysis

**Figure 3:**
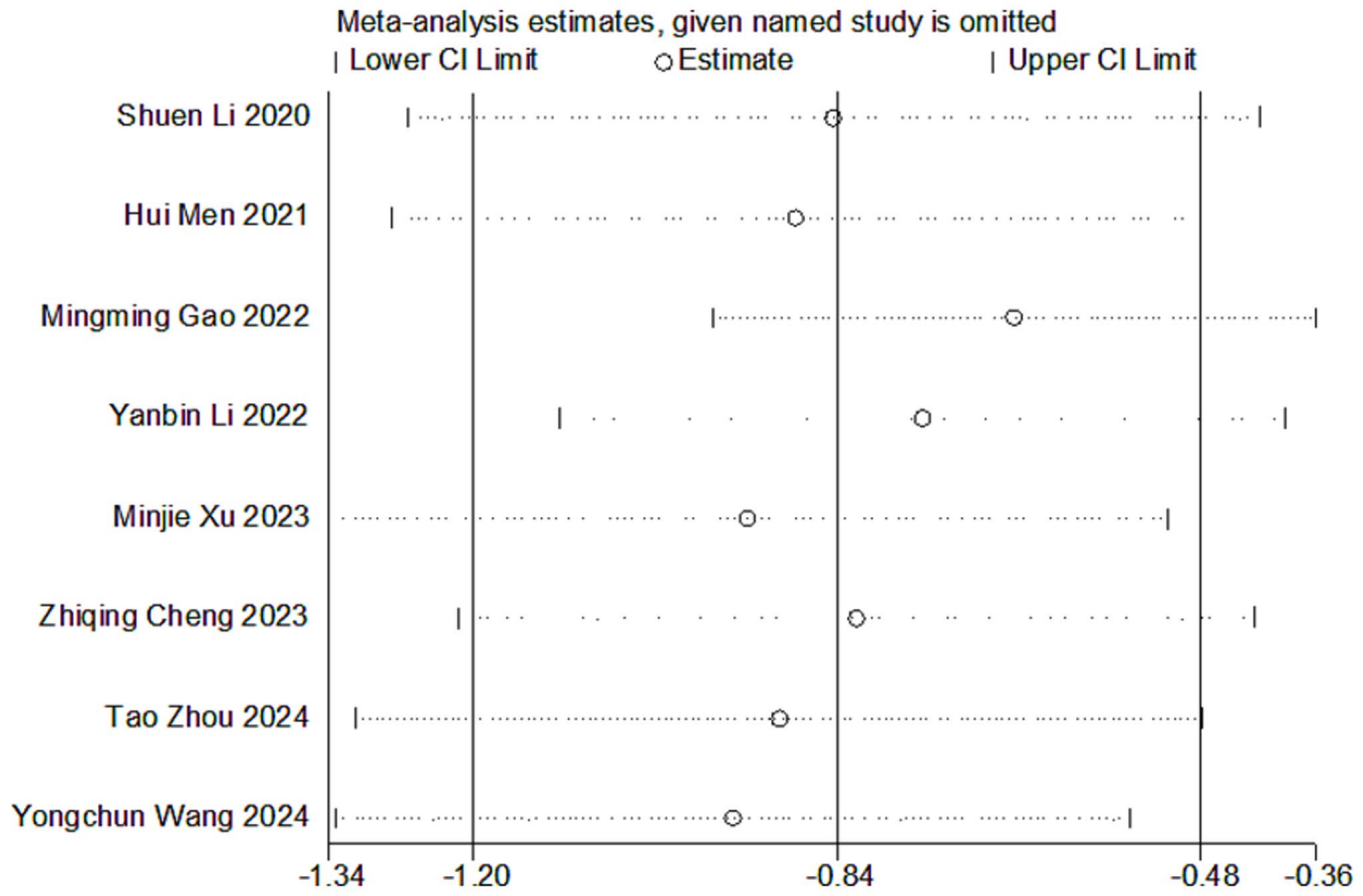
Sensitivity analysis of serum albumin level.

We assessed the stability of the pooled results for serum albumin levels (Figure 3) by sensitivity analysis. The results showed that removing individual studies did not significantly alter the pooled results (Supplementary Table S3).

## 4 Discussion

We carried out this meta-analysis to evaluate the predictive role of serum albumin levels and albumin-related nutritional indices for PSCI. There were nine studies involving 2,332 stroke patients included. Our meta-analysis showed that serum albumin levels, GNRI scores, and HALP scores were significantly lower in the PSCI patients compared to the PSNCI.

A rising number of studies have suggested that malnutrition may lead to cognitive deficits after stroke (25, 26). Firstly, malnutrition can impair the neuroplasticity capacity, so that it reduces the ability to repair damaged brain tissue, which is crucial for the recovery of cognitive function (27, 28). In addition, poor nutrition can exacerbate inflammation and oxidative stress after a stroke, resulting in further damage to brain cells and cognitive function (29). Therefore, paying close attention to the nutritional status of stroke patients is essential for the prevention and intervention of PSCI.

Serum albumin, as one of the markers of nutritional status, is not only involved in maintaining fluid balance and facilitating nutrient transport, but also has important immunomodulatory functions (8). Wu et al. (30) revealed that the elderly population with cognitive impairment had lower serum albumin levels. In recent years, nutritional indices related to albumin have received extensive attention, such as GNRI scores and HALP scores.

Once a stroke occurs, a great deal of free radicals will be produced in the brain due to the lack of oxygen. Free radicals peroxidize macromolecules such as DNA, lipids, and proteins, causing irreversible damage and leading to severe cellular damage (31). Besides, the reaction between free radicals and neurotransmitters may lead to the production of endogenous neurotoxins, possibly leading to cognitive defects (32). Serum albumin has an antioxidant activity that could remove the massive free radicals produced after stroke, thereby reducing the adverse effects of oxidative damage (33). With further research, it has been gradually discovered that albumin has a regulatory role in the central nervous system. Xie Yi et al. (34) have shown that albumin can moderate astrocyte and microglia activity by combining with Aβ proteins, reducing the production of tau proteins and aggregation of tubulin, as well as reducing neuronal loss and synaptic alterations (35, 36). In addition, Wang et al. (37) have found that serum albumin may reduce the blood–brain barrier permeability, delay vascular endothelial damage due to cerebral ischemia, and maintain neuronal activity. Therefore, when serum albumin levels decline, the risk of cognitive impairment will increase.

Previously, assessment of the nutritional status of stroke patients was often accomplished in the form of scales. However, such structured scales are difficult for stroke patients to complete accurately (38–40). As a nutritional index that combines serum albumin levels and body metrics, GNRI has been used in the prognosis of patients with cardiovascular disease and cancer due to its objectivity, accuracy, and easy accessibility (41, 42). Minwoo Lee et al. (20) concluded that lower GNRI scores were independently associated with PSCI, which is consistent with our findings. However, Wang et al. (8) have not found a statistical difference in GNRI scores between PSCI and PSNCI patients. This probably resulted from different inclusion criteria for patients and sample sizes. There is a need for larger sample sizes, multiple-center studies in the future to explore the relationship between GNRI and PSCI.

The HALP score, combining hemoglobin, albumin, lymphocyte, and platelet, has been suggested as a simple measure of systemic inflammation and nutritional status. In terms of inflammation, lymphocytes and platelets have been shown to be factors that exacerbate ischemic brain injury and neurological damage (43). Lymphocytes have tissue repair and neuroprotective effects. Studies have indicated that lymphocytes exert neuroprotective functions by generating anti-inflammatory factors so as to suppress the process of inflammation (44). Nevertheless, stroke patients always have a reduced quantity of lymphocytes, which prevents the repair of injury after stroke and promotes the development of PSCI (45). Platelets are also involved in the regulation of immunity and inflammation after stroke (46). After a stroke occurs, platelets will be activated, causing leukocytes to enter damaged tissues and triggering further inflammatory events (24). In terms of nutrition, similar to albumin, hemoglobin is also an important indicator of the nutritional status of the body. Decreased hemoglobin can reduce the ability of the brain to deliver oxygen, which may lead to mitochondrial disease and neuronal damage (20, 47). A study has shown a correlation between low HALP scores and poor outcomes in patients with acute ischemic stroke (48). Besides, Zhou et al. (24) and Xu et al. (10) showed that HALP scores correlate with PSCI, which is helpful for early identification of people at high risk of PSCI, this is consistent with our meta-analysis.

We performed subgroup analyses to identify factors contributing to heterogeneity. Firstly, subgroup analyses in this study showed that both prospective cohort studies, as well as retrospective cohort studies and case–control studies, suggested that serum albumin was significantly lower in patients with PSCI. Secondly, serum albumin levels were significantly associated with PSCI in the MMSE group, whereas this relationship was not significant in the MoCA group. This suggests that differences in cognitive assessment tools may be one of the factors contributing to the high heterogeneity. Additionally, in terms of blood testing time, the results of the ≤ 48-h blood testing group showed a significant association between reduced serum albumin and PSCI. Meanwhile, we also found this association in the subgroup analysis of the time after stroke onset in both acute (≤72 h) and early subacute phases (≤1 week). These results suggest that lower albumin levels are not only a marker of acute stress, but may be involved in the early course of PSCI through persistent pathologic mechanisms. However, the results of subgroups containing only one study (MMSE+MoCA group, late subacute group) and subgroups with unlimited time need to be interpreted with caution.

This study also has some limitations. Firstly, because of the small number of original studies, certain subgroups consisted of only 2 studies, which limits statistical validity. Of the nine included studies, only one was from Korea, and the remaining eight were from China, which would limit the generalizability of our findings. Secondly, the language of the included literature was limited to English and Chinese, which may make our findings have selection bias. Finally, despite subgroup analyses, there was consistently high heterogeneity among the subgroups, suggesting that the high heterogeneity was due to multiple factors. We performed a methodological review of the original studies and found that cognitive assessment thresholds may be a factor contributing to the high heterogeneity, such as the study by Zhou et al. (24) which included patients with MoCA < 23 points as the PSCI group, whereas Cheng et al. (23) categorized patients with MoCA < 26 points as the PSCI group. Furthermore, the stroke severity may be another important factor influencing the heterogeneity, as Li et al.’s (18) study included only patients with mild stroke, whereas the patients included in Gao et al.’s (21) study had a higher stroke severity. Future studies should focus on standardizing time reporting, exploring the trajectory of albumin dynamics, and prospectively evaluating the potential value of early correction of low albumin in preventing PSCI.

## 5 Conclusion

In conclusion, decreased serum albumin levels and albumin-related nutritional indices (GNRI and HALP scores) were significantly associated with PSCI. However, due to regional selection bias and the small number of original studies, future research involving larger samples is necessary to verify the predictive value of albumin-related nutritional indicators for PSCI further.

## Data Availability

The original contributions presented in the study are included in the article/Supplementary material, further inquiries can be directed to the corresponding author.
